# Cost-Effectiveness Analyses on Various Models of The Red Light, Purple Light Self-Regulation Intervention for Young Children

**DOI:** 10.3389/fpsyg.2021.711578

**Published:** 2021-10-14

**Authors:** Tao Li, Megan M. McClelland, Shauna L. Tominey, Alexis Tracy

**Affiliations:** ^1^Health Management and Policy, Oregon State University, Corvallis, OR, United States; ^2^Human Development and Family Sciences and the Hallie E. Ford Center for Healthy Children and Families, Oregon State University, Corvallis, OR, United States; ^3^Extension Family and Community Health and the Hallie E. Ford Center for Healthy Children and Families, Oregon State University, Corvallis, OR, United States

**Keywords:** cost-effectiveness analysis, economic evaluations, red light purple light, early childhood intervention, self-regulation, school readiness, executive function skills

## Abstract

Early childhood interventions can improve self-regulation, but there are few economic evaluations of such interventions. This study analyzed the cost-effectiveness of an early childhood self-regulation intervention (*Red Light Purple Light!*; RLPL), comparing three different models of implementation across stages of intervention development: (Model 1) trained research assistants (RAs; graduate students) directly delivered the RLPL intervention to children; (Model 2) RAs trained trainers (e.g., program coaches), who then trained teachers to implement RLPL with children (e.g., train-the-trainer); and (Model 3) program faculty trained teachers to deliver the RLPL intervention to children. We implemented a cost-effectiveness analysis by calculating the incremental cost-effectiveness ratio. We also conducted a series of sensitivity analyses to adjust for parameter uncertainty. Our base-case analysis suggests that Model 2 was the most cost-effective strategy, in that a cost of $23 per child was associated with a one-unit increase of effect size on self-regulation scores. The “train-the-trainer” model remained the optimal strategy across scenarios in our sensitivity analysis. This study fills an important gap in cost-effectiveness analyses on early childhood self-regulation interventions. Our process and results can serve as a model for future cost-effectiveness analyses of early childhood intervention programs and may ultimately inform decisions related to intervention adoption that optimize resource allocation and improve program design.

## Introduction

Many children, especially those experiencing socio-demographic risks such as poverty, enter formal schooling without key skills needed to thrive in a classroom environment (Blair and Raver, 2015). These skills are included in a construct called *school readiness* and include self-regulation and early academic skills (Snow, 2006). Unfortunately, children facing socio-demographic risk who struggle with self-regulation and early academic skills are likely to face achievement gaps that persist and widen over time (Zelazo et al., 2016). Self-regulation is a significant predictor of short- and long-term academic, social, and life outcomes (Moffitt et al., 2011; McClelland et al., 2013; Zelazo et al., 2016; Robson et al., 2020). Importantly, self-regulation can be practiced and improved (Diamond and Ling, 2016), and self-regulation interventions may serve as a mechanism to protect children at risk (Sasser et al., 2017; Pandey et al., 2018). Large-scale classroom curricula that combine self-regulation and academic skills have shown mixed effects (Farran et al., 2013; Blair and Raver, 2014; Morris et al., 2014), but targeted self-regulation interventions that can easily be implemented in the classroom have shown positive effects across both self-regulation and academic domains (Tominey and McClelland, 2011; Schmitt et al., 2015; McClelland et al., 2019).

*Red Light Purple Light!* (RLPL) is an easy-to-implement, short-term intervention designed to promote self-regulation and school readiness (Tominey and McClelland, 2011; McClelland and Tominey, 2015). Although early childhood interventions such as RLPL have been shown to improve self-regulation, there are few economic evaluations of such interventions. This study analyzed the cost-effectiveness of an early childhood self-regulation intervention (RLPL) implemented through three different models: 1) trained research assistants (RAs) implemented the RLPL intervention with children; 2) RAs trained trainers (e.g., early childhood coaches), who then trained teachers to implement the RLPL intervention with children (e.g., train-the-trainer); and 3) program faculty trained teachers to implement RLPL with children. These three models used the same intervention curriculum, but at different phases of testing and iterative development of the implementation process, which allowed us to explore and compare the cost-effectiveness of each implementation model.

### The Importance of Self-Regulation on School Success

Self-regulation is a complex construct that includes aspects of emotion, cognition, and behavior (McClelland et al., 2010). This paper focuses on the aspects of self-regulation most relevant for children’s learning in school contexts, which stem from three executive function (EF) processes: working memory, attentional or cognitive flexibility, and inhibitory control (Cameron Ponitz et al., 2009). Working memory refers to the ability to hold and manipulate short-term instructions in mind such as when children need to remember the steps in an art activity (Gathercole, 2008; McClelland et al., 2015); attentional or cognitive flexibility is ability to focus on a task and switch to a new task when needed such as when children need to stop what they are doing and move to a new activity (Rothbart and Posner, 2005); and inhibitory control is the ability to stop a dominant response in favor of a more adaptive one (Blair, 2003). This can be seen when children have to inhibit their impulse to blurt out an answer and raise their hand instead. Although each aspect of EF has been shown to predict academic outcomes, their integration is particularly important for school success (McClelland et al., 2007b, 2014; McClelland and Cameron, 2012). We use the term self-regulation in the present study to capture EF processes in real-world settings.

#### Connections Between Self-Regulation and Academic Skills

Self-regulation is a key factor supporting academic success across the life span (McClelland et al., 2006, 2013; Blair and Razza, 2007; Moffitt et al., 2011). Self-regulation predicts early achievement (both math and literacy) in preschool, elementary and middle school and even college (Duckworth et al., 2010; McClelland et al., 2013, 2014). Especially important for school readiness skills, self-regulation is related broadly to early aspects of math and emergent literacy (McClelland et al., 2007a, 2014; Schmitt et al., 2017), especially early numeracy skills (counting, cardinality, numeral knowledge), which is most predictive of later mathematics achievement (Nguyen et al., 2016). Moreover, self-regulation interventions have shown significant effects on children’s math and literacy (Blair and Raver, 2014; Schmitt et al., 2015; Pandey et al., 2018) and may be especially predictive of early math skills (Allan et al., 2014; McClelland et al., 2014; Blair et al., 2015; Purpura et al., 2017).

In previous studies evaluating the RLPL program, effects have been found for children’s improvements in self-regulation (Schmitt et al., 2015; Duncan et al., 2018), especially those with low baseline levels of self-regulation (Tominey and McClelland, 2011), early math skills (Duncan et al., 2018), particularly for low socioeconomic (McClelland et al., 2019) and low-SES DLL children (Schmitt et al., 2015) and early literacy skills (Tominey and McClelland, 2011; Duncan et al., 2018). These effects are supported by other research showing that classrooms characterized by consistent, organized classroom practices lead to better academic outcomes for children (Cameron et al., 2008; Cameron and Morrison, 2011). Previous research on RLPL also indicates that participating children demonstrate significantly stronger self-regulation on direct measures and teacher ratings (Schmitt et al., 2015; Keown et al., 2020) suggesting that children are demonstrating improvements in directly assessed self-regulation and observations of children’s behavior in classroom settings. These results are promising but do not provide information related to the costs associated with each model or the corresponding effects, indicating that an evaluation of the cost-effectiveness of the RLPL program is needed.

### The Potential of Early Interventions

Early childhood is an important developmental period for self-regulation growth because of the rapid development in brain development, especially in the prefrontal cortex, an area that is critical for self-regulation (Diamond, 2002; Montroy et al., 2016). As such, early childhood settings provide an opportune time for interventions aimed at strengthening these skills in young children. Research shows that significant differences in self-regulation are apparent in early childhood (McClelland et al., 2015; Zelazo et al., 2016). Although some of these differences in self-regulation are likely on account of natural variability in children’s developmental ages and stages, these differences relate to children’s abilities to engage in positive classroom behaviors (Day et al., 2015; Zelazo et al., 2016). Research on the high rates of preschool suspensions and expulsions indicates that early childhood teachers could benefit from support managing challenging classroom behaviors likely related to self-regulatory difficulties (Gilliam and Shabar, 2006). Early childhood educators enter the field with a range of educational backgrounds and experiences, making in-service trainings an especially valuable form of professional development for the early childhood field. Professional development is seen as a critical component of high-quality early childhood education settings, and thus, significant time and funds are devoted to supporting these efforts. It is through ongoing professional development that interventions, including self-regulation interventions, are often implemented.

Interventions focused on the broader construct of social–emotional skills (Diamond et al., 2007; Domitrovich et al., 2007) have emerged in recent years, including programs specifically targeting self-regulation (e.g., Tominey and McClelland, 2011; Schmitt et al., 2015). Numerous programs have demonstrated significant positive effects on children’s self-regulation, although effects are typically modest (Bierman et al., 2008; Raver et al., 2011; McClelland et al., 2017). In some cases, program effects have even been inconsistent across studies (Blair and Raver, 2014; Morris et al., 2014). The resources, time, and skills required to implement early childhood intervention programs to fidelity vary widely and are not often well-documented. Few interventions have conducted cost-effectiveness analyses to identify the costs associated with implementation as well as associated outcomes. Conducting cost-effectiveness analyses is a critical next step in the field of early childhood intervention to inform practice and policy toward identifying programs that are the most feasible and cost-effective.

### The Importance of Cost-Effectiveness Analysis in Early Intervention Programs

There is a growing interest among researchers, practitioners, and policymakers in studying the economic impacts of early childhood interventions to inform investment and policy decisions (National Academies of Sciences, Engineering and Medicine, 2016; Cannon et al., 2018; Jones et al., 2019). Although few studies exist for school readiness interventions, researchers have started to examine costs more closely. For example, Jones and colleagues conducted cost analysis and examined the costs of different versions of the Head Start Research-based, Developmentally Informed intervention (Jones et al., 2019). Compared to cost analysis, we expect cost-effectiveness analysis will be a more powerful tool and will provide more important evidence to inform policymakers and stakeholders, because in cost-effectiveness analysis we simultaneously take into account the resources needed for program implementation as well as the outcomes achieved. Thus, to echo this urgent need of economic evaluation evidence, the purpose of this study is to evaluate the cost-effectiveness of the RLPL program.

### The Present Study

The present study focuses on a cost-effectiveness analysis of the *Red Light, Purple Light* (RLPL) self-regulation intervention, which has been shown to have significant positive effects on self-regulation (e.g., Tominey and McClelland, 2011; Schmitt et al., 2015; Duncan et al., 2018; McClelland et al., 2019). The RLPL program is an eight-week classroom-based self-regulation intervention. The program consists of large group time music and movement-based classroom games implemented twice a week in 20–30-min sessions (McClelland and Tominey, 2015). Over the course of multiple randomized control trials, the same RLPL curriculum was used, although the training and implementation models varied. This variation allowed for an exploratory cost-effectiveness analysis across each of these models.

RLPL was developed through an iterative development process. The intervention was first piloted through a small randomized trial where the sessions were led by a research assistant (RA) who was also a former early childhood classroom teacher (Model 1; Tominey and McClelland, 2011). The intervention was replicated in another randomized trial with a larger sample with RAs leading the games during classroom group time activities (also Model 1; Schmitt et al., 2015). A “train-the-trainer” model was then employed (Model 2; Duncan et al., 2018) where two early childhood professional development coaches were trained in the intervention by the RLPL development team and then delivered the training to additional teachers in their district as part of their existing role supporting professional development efforts. Finally in Model 3, professional development trainings were delivered to teachers by faculty members of the original RLPL development team (McClelland et al., 2019).

In the present cost-effectiveness study, cost and effectiveness data are analyzed for each approach to implement and to identify the model(s) that are most cost-effective. Although the RLPL program was implemented across studies using various models, each shared the common goal of improving children’s self-regulation. Therefore, these data provide a unique opportunity to compare cost-effectiveness among different models of the RLPL intervention, as the cost-effectiveness analysis is particularly helpful to compare among closely related interventions (Li et al., 2017, 2019). We expect our findings will help identify the optimal intervention design and strategy to improve children self-regulation in the most cost-effective way, and will provide critical evidence to inform decision making related to intervention selection for schools, districts, and additional stakeholders.

## Materials and Methods

### Participants

Data included in this study were collected as part of four intervention studies examining the RLPL intervention in young children. The four studies fit three models of RLPL implementation as described below (see Table 1). Below, we describe the original samples and in the analytic strategy section we detail how sample sizes were analyzed to improve comparability across models. The study was approved by the Institutional Review Board at Oregon State University, United States.

**Table 1:** Parameters Sources and Values for Base-case Analysis on Different Models to Implement the RLPL Program.

RLPL implementation model^a^	Model 1	Model 2	Model 3
Literature source	Tominey and McClelland, 2011; Schmitt et al., 2015	Duncan et al., 2018	McClelland et al., 2019
*N (children)*	65; 276	125	157
Number of classrooms in intervention^b^	16	4	10
Child gender (% female)	60%/51%	N/A^c^	52%
Child age (in months)	54.60/51.69	63.24	51
Low-income	50%/100%	N/A	100%
English language learner	4.6%/33%	N/A	33%
Average parent education level	14.6years (Associate’s Degree equivalent)/11.5years (less than High School)	N/A	11.27years (less than High School equivalent)
Total person-hours spent in RLPL implementation	62	6	6
Dosage^d^	16 sessions (*M*= 6.67h)	15 sessions (*M*= 6.25h)	16 sessions (*M*= 6.67h)
Effect size of self-regulation scores	0.34/0.34	0.34	0.31

a Model 1=trained research assistants (RAs) directly delivered the RLPL games to children; Model 2=trained RAs trained trainers, who then trained teachers to deliver the RLPL games to children; Model 3=program faculty trained teachers, who then delivered the RLPL games to children.

b There were 16 classrooms that received RLPL intervention under Model 1 (9 in the Tominey and McClelland, 2011 study, and 7 in the Schmitt et al., 2015 study), and 10 classrooms in intervention under Model 3 (McClelland et al., 2019). In Model 2, RAs trained trainers, who then trained a total of 4 teachers to deliver the RLPL games, so we analyzed cost for 4 classrooms.

c N/A=Not available.

d For Models 1 and 3 dosage=(25min per session ^*^ 16 sessions). For Model 2 dosage=(25min per session ^*^ 15 sessions).

### Model 1: Trained Research Assistants (RAs; Graduate Students) Deliver the RLPL Games to Children

#### Tominey and McClelland (2011)

Sixty-five children (50% low-income as defined as participation in Head Start) from nine classrooms in two preschool centers participated in the study (children were randomized within classrooms). Children were an average of 54.60months (range: 44–60months; 60% female). The average maternal education level was about an Associate’s degree (*M*= 14.6years) with a range of 6–21years. Three children spoke Spanish as a first language, were assessed in Spanish, and received the intervention in Spanish.

#### Schmitt et al. (2015)

Two hundred seventy-six children from 14 Head Start classrooms across nine preschool centers participated (7 intervention and 7 control classrooms). Children averaged 51.69months (range: 37.98 to 66.04months; 51% female). The average parent education level was less than a high school degree (*M*=11.15years) with a range from 2 to 16years of education. There were 33% (*N*=88) English language learners (ELLs) who were assessed in Spanish and who received the intervention in Spanish.

### Model 2: RAs Train Trainers, Who Then Train Teachers to Deliver the RLPL Games to Children (“Train-the-Trainer”)

#### Duncan et al. (2018)

In this implementation model, one hundred twenty-five children from four schools in a large public school district participated in the study with a final number of 99 children with data at both time points. Children were an average age of 63.24months and did not have prior preschool experience. No additional demographic information (i.e., parent education level) was available from the school district. All children received the RLPL intervention and self-regulation assessment in English.

### Model 3: Program Faculty Train Teachers, Who Then Deliver the RLPL Games to Children

#### McClelland et al. (2019)

One hundred fifty-seven children (52% female) from 13 Head Start classrooms participated in the study (10 intervention, 3 control). Children were an average of 51months (range of 38–62months), and parents’ education level ranged from 2 to 17years (M=11.27). Fifty-eight percent of the sample identified as Latino and 33% (*N*=62) were ELLs. These children were assessed in Spanish but received the intervention in English following best practices for supporting Dual Language Learners (DLL’s; Tominey and O'Bryon, 2018).

### Procedure

Across the four studies, the RLPL intervention was implemented in three different models as outlined below. In all studies, teachers reported that their normal curricula did not include games or activities that were similar to the RLPL games or emphasized self-regulation. Importantly, for all models in our study, participation in the intervention (versus a delayed control) was randomized. Individuals involved in data collection were blind to the condition of participating children and classrooms where randomization occurred. Children in all delivery models received similar amounts of the intervention. For Models 1 and 3, the duration was twice a week over eight weeks for 16 sessions, and in the Model 2, the duration was five days a week over three weeks for 15 sessions. Each session was 20–30min across all delivery models. On average, the dosage was 6.67h for Models 1 and 3 (average of 25min per session x 16 sessions), and 6.25h for Model 2 (average of 25min per session x 15 sessions; see Table 1).

#### Model 1:

In this implementation model, RLPL was administered by trained RAs who pulled children out of their classroom to implement the RLPL games and activities.

##### Tominey and McClelland (2011)

Children within nine classrooms were randomly assigned to receive the intervention (*n*=28) or to a Business-As-Usual (BAU) control group (*n*= 37; overall *N*=65). Trained RAs assessed children’s self-regulation individually, and parents and teachers completed demographic surveys. Children were pretested in the fall, the intervention was administered in the winter by RAs, and children were post-tested in the spring of the preschool year.

##### Schmitt et al. (2015)

Fourteen classrooms were randomly assigned to an intervention group (*n*=7; 126 children) or a BAU control group (*n*=7; 150 children; *N*=276 overall). Trained RAs directly assessed children’s self-regulation in the fall (pre-test) and in the spring (post-test) of the preschool year. The intervention was implemented in the winter by RAs. Parents and teachers completed demographic surveys.

#### Model 2:

##### Duncan et al. (2018)

In this “train-the-trainer” model, RAs trained trainers who then trained teachers to deliver the RLPL games to children in a summer program. Of the participating schools, three schools were randomly assigned to receive the intervention (*N*= 67 children) and one school was randomly assigned to serve as a control group (receiving a summer program curricula, which was different from RLPL; *N*=32). Teacher fidelity and feasibility surveys indicated that teachers implemented the games as intended and children enjoyed playing them. Children’s self-regulation was directly assessed at pre-program (July) and post-program (August) time points by trained school district staff.

#### Model 3:

##### McClelland et al. (2019)

In this model, program faculty trained teachers in the standard three-hour RLPL training, who then delivered the RLPL games to children. Classrooms were randomly assigned to two versions of the RLPL intervention (*N*=10 classrooms total; 120 children) or a BAU control group (*N*=3 classrooms; 37 children). In the fall (pre-test) and spring (post-test) of the preschool year, direct assessments of self-regulation were administered in English or Spanish. The intervention was implemented in the winter by teachers in either English or Spanish. Teacher fidelity and feasibility surveys demonstrated that teachers implemented the intervention with high fidelity. Parents and teachers completed demographic questionnaires.

### Outcomes

#### Self-Regulation

To assess children’s self-regulation, the Head-Toes-Knees-Shoulders (HTKS) measure was used in all studies. The HTKS assesses aspects of executive function including attention, working memory, and inhibitory control (McClelland et al., 2014) in children ages 3–8. The task has three parts. In the first part, children are asked to touch their head (or toes) when asked to touch their toes (or head). In the next part, a new rule is introduced where children touch their knees (or shoulders), and then, both rules are included (head/toes opposite and knees/shoulders opposite). In the third part, children are still doing the opposite, but the rules are switched (head goes with knees and shoulders goes with toes). Items are scored 0 for an incorrect response, 1 for a self-corrected response, and 2 for a correct response. The overall scores range from 0 to 60 except in Tominey and McClelland (2011) when the task had two parts and scores ranged from 0 to 40.

In the McClelland et al. (2019) study (Model 3 above), children received a revised version of the HTKS, called the HTKS-Revised (Gonzales et al., 2021). This version included a downward extension with the same commands where children responded to using verbal cues (e.g., “When I say Toes, you say Head”) rather than gross motor responses. This section was administered to children prior to receiving parts 1–3 of the original HTKS measure and overall scores ranged from 0 to 116. The HTKS-R and HTKS have both demonstrated strong reliability and validity in preschool aged children (McClelland et al., 2014; Gonzales et al., 2021). Previous research has demonstrated that the HTKS is sensitive to intervention effects, where children receiving an intervention showed significant improvement in self-regulation when compared with children in a control group (Tominey and McClelland, 2011; Schmitt et al., 2015; Duncan et al., 2018; Landis et al., 2018; Upshur et al., 2019).

### Analytic Strategy for the Cost-Effectiveness Analysis

We conducted our cost-effectiveness analyses from the perspective of RLPL program implementation (i.e., the costs associated with conducting research were not included as these expenses were supported by grants and would not be included in future implementation and program costs). In doing so, we reduced parameter uncertainty in our data collection; our analyses were more directly relevant to program implementation; and our findings could better support stakeholders’ decisions on replicating and expanding our intervention. Based on this analytical perspective, we included costs accrued from intervention implementation only, excluding costs accrued for research and evaluation purposes. We applied the concept of opportunity cost when estimating cost, which could better identify the trade-off value of cost items. As recommended by the Panel on Cost-Effectiveness in Health and Medicine, “(a) change in the use of a resource caused by a health intervention should be valued at its opportunity cost, which is the value the resource could have produced if it were spent in its best available alternative use” (Weinstein et al., 1996, p. 1255). We considered program cost as the total cost to implement the RLPL program, which include personnel cost and material cost. Regarding personnel cost, we excluded teachers’ time spent in receiving trainings (e.g., trainers’ time in Model 2) and their time spent in delivering the RLPL games in classroom (e.g., teachers’ time in Models 2 and 3) during their regular work hours from our cost estimation, because these teachers would have to spend such time on work even without the RLPL program. For faculty, staff, and graduate students in our RLPL implementation team, we calculated their personnel costs as the product of their wages and their time spent in either training the teachers (e.g., RAs’ and faculty’s time in Models 2 and 3) or directly delivering the intervention (e.g., RA’s cost in Model 1). The RLPL program coordinator recorded the wages and time spent information for faculty, staff, and graduate students in each model. In addition to personnel cost, the intervention cost also includes $100 per classroom materials kit to deliver the RLPL games in each classroom. Classroom material kits included items currently found in early childhood classrooms for use during the intervention games (e.g., different colored circles cut out of construction paper, classroom drum and musical instrument egg shaker set). After estimating total program cost equal to the sum of total personnel cost and total material cost, we divided total program cost by the number of students to get the average intervention cost per student for each model.

Table 1 presents the parameters sources and values used in our base-case analysis. According to previous research, the RLPL program had been implemented according to three models:

Model (1) RAs directly delivered the RLPL games to children (Tominey and McClelland, 2011; Schmitt et al., 2015);

Model (2) RAs trained trainers, who then trained teachers to deliver the RLPL games to children (Duncan et al., 2018); and.

Model (3) program faculty trained teachers, who then delivered the RLPL games to children (McClelland et al., 2019).

We extracted data from previous research to assess both cost and effectiveness for each model. There were 16 classrooms that received RLPL intervention under Model 1 (9 in the Tominey and McClelland, 2011 study, and 7 in the Schmitt et al., 2015 study), and 10 classrooms in intervention under Model 3 (McClelland et al., 2019). In the “train-the-trainer” model, RAs trained trainers, who then trained a total of 4 teachers to deliver the RLPL games, so we calculated cost of Model 2 for 4 classrooms (Table 1). As the typical class size for early childhood education settings is 15–20 children (specific to programs included in these studies), in our base-case analysis we standardize our estimates as 18 children in each classroom. This standardization is important to improve comparability across models, and can better support stakeholders’ decisions on replicating and expanding the RLPL programs. We then calculated the average intervention cost per student. Aligned with the goal of the RLPL program to improve children self-regulation, we used the effect size of self-regulation scores to measure effectiveness.

**Figure 1:**
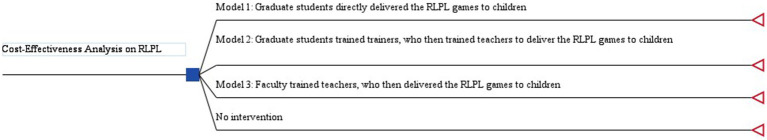
Cost-effectiveness Analytical Model.

Figure 1 illustrates our cost-effectiveness analytical model. We evaluated cost-effectiveness among the three models of the RLPL program and compared that to no intervention. To conduct the cost-effectiveness analysis, we calculated the incremental cost-effectiveness ratio (ICER) associated with the RLPL program. Therefore, we would interpret our cost-effectiveness findings as the cost of additional investment in our intervention to gain one additional unit of outcome improvement.

In addition to base-case analysis, we conducted a series of sensitivity analyses to adjust for parameter uncertainty. This would not only test the robustness of our main findings, but also provide critical evidence to inform decision makings on adopting and expanding our interventions at various scales and under different scenarios. Specifically, we conducted sensitivity analyses under three scenarios:

Scenario (1) In our base-case analysis, we assumed 18 children in each classroom. The typical class size for early childhood education settings included in the present study is 15–20 children. We ran a sensitivity analysis to adjust the number of children per classroom from 15 to 20 for each model.

Scenario (2) We ran a sensitivity analysis to adjust the potential variance in workload to implement the intervention, by increasing and reducing person-hours by 20% for each model.

Scenario (3) In our base-case analysis we did not include facility cost, because such cost would still exist even without our RLPL program. Research suggests this cost could range from 10 to 20% of total cost (Jones et al., 2019). We ran a sensitivity analysis to adjust for facility cost, and we increased the total cost by the upper limit of 20% for each model. This allowed us to draw a more conservative cost-effectiveness results, and could comprehensively adjust for various uncertainties of increasing cost.

We used TreeAge Pro software (TreeAge Pro 2021, Healthcare R2, 2020) to implement our analyses. We adjusted all monetary values to 2019 dollars by consumer price index (Bureau of Labor Statistics, 2020).

## Results

Our base-case cost-effectiveness results are shown in Table 2. Although the program effectiveness was similar across the RLPL models, the cost varied from $7.72 per child in Model 2 to $11.15 per child in Model 1. Our base-case analysis suggested that Model 2 was the most cost-effective strategy with an ICER of 22.71. Other RLPL models were dominated, as they were associated with lower or same effectiveness at a higher cost compared to Model 2.

**Table 2:** Cost-effectiveness Results in Base-case Analysis.

Cost-effectiveness ranking^a^	Cost ($ per child)^b^	Incremental cost	Effectiveness (effect size of self-regulation scores)	Incremental effectiveness	Incremental cost-effectiveness ratio
No intervention	0	–	0	−	
Model 2	7.72	7.72	0.34	0.34	22.71
Model 3	7.85	0.12	0.31	−0.03	dominated
Model 1	11.15	3.43	0.34	0	dominated

a Cost-effectiveness analysis order is from the lowest-cost strategy to the highest-cost strategy. Model 1=trained research assistants (RAs) directly delivered the RLPL games to children; Model 2=trained RAs trained trainers, who then trained teachers to deliver the RLPL games to children; Model 3=program faculty trained teachers, who then delivered the RLPL games to children.

b Monetary values were adjusted to 2019 dollars by consumer price index.

Table 3 shows the results under various scenarios in our sensitivity analysis. Model 2 was consistently the most cost-effective strategy across Scenarios 1 to 3, while Models 1 and 3 were dominated. Specifically, the ICER of Model 2 decreased from 27.25 to 20.44 when we adjusted the number of children per classroom from 15 to 20 (Scenario 1), increased from 21.44 to 23.99 when we adjusted the variance of person-hours from 80 to 120% (Scenario 2), and increased from 22.71 to 27.25 when we adjusted the total implementation cost from 100 to 120% (Scenario 3).

**Table 3:** Cost and Incremental Cost-effectiveness Ratio (ICER) of Model 2 Under Various Scenarios in Sensitivity Analysis.^a^

Scenario 1: Adjust number of children per classroom		
Number of Children per classroom	Cost per child ($)	ICER
15	9.27	27.25
16	8.69	25.55
17	8.18	24.05
18 (Base case)	7.72	22.71
19	7.32	21.52
20	6.95	20.44
**Scenario 2: Adjust person-hours to implement interventions**		
**Percentage change of person-hours**	**Cost per child ($)**	**ICER**
−20%	7.29	21.44
−10%	7.51	22.08
Base case	7.72	22.71
+10%	7.94	23.35
+20%	8.16	23.99
**Scenario 3: Adjust total cost to implement interventions**		
**Percentage change of total cost**	**Cost per child ($)**	**ICER**
Base case	7.72	22.71
+5%	8.11	23.85
+10%	8.49	24.98
+15%	8.88	26.12
+20%	9.27	27.25

a Only shows results of Model 2 (the “train-the-trainer” model) compared to no intervention in sensitivity analysis, because other models were dominated.

## Discussion

The present study conducted cost-effectiveness analyses on three implementation models of the *Red Light, Purple Light* (RLPL) intervention, a self-regulation intervention for young children. The three implementation models included: (Model 1) trained RAs directly delivered the RLPL intervention to children; (Model 2) RAs trained trainers (e.g., program coaches), who then trained teachers to implement RLPL with children (e.g., train-the-trainer); and (Model 3) program faculty trained teachers to deliver the RLPL intervention to children. Results of base-case analysis indicated that Model 2 was the most cost-effective strategy. We estimated that by implementing the “train-the-trainer” model, a cost of $23 per child was associated with a one-unit increase of the effect size of self-regulation scores. Our base-case finding remains robust to a series of sensitivity analyses, when we adjusted the number of students per classroom, the variance of person-hours, and the total cost to implement the RLPL program. The “train-the-trainer” model remained the optimal strategy across these scenarios, with ICER ranging from 20 to 27.

### Iterative Intervention Development

The present study was unique in several respects. Notably, having access to data from randomized control trials where the same curriculum was tested using different models of implementation allowed for comparison of cost-effectiveness across these models. Each model was implemented at a different point in time and different stage of the iterative development process for the RLPL intervention. Although the intervention curriculum remained the same across implementation methods, the methods of implementation changed over time. In the first study, the intervention was delivered by the curriculum developers. This model is critical as an initial step in testing a new intervention, but not feasible to support long-term scaling efforts. The models that followed shifted the intervention from the developer to other trained individuals (research assistants and faculty members with experience in early childhood) and finally to teachers. The second and third models (RAs training trainers who then trained teachers and program faculty training teachers) were only possible in later iterations of the intervention, but also reflected a feasible method of intervention delivery and implementation in current early childhood contexts.

### The Importance of Cost-Effectiveness Studies in School Readiness Interventions

In the search to support children’s school readiness and success, a plethora of early childhood interventions have been developed and tested (Blair and Raver, 2014; Sasser et al., 2017; Diamond et al., 2019; Welsh et al., 2020). Although research supports the effectiveness of these interventions, it is unclear if they are cost-effective for early childhood programs to implement and few programs have been evaluated to examine this. Cost-effectiveness analyses can provide important evidence to support decision makings of investing in interventions and optimizing program designs. However, the literature on cost-effectiveness analyses of early childhood self-regulation intervention has been sparse. Corso and colleagues (Corso et al., 2015) conducted cost-effectiveness analyses on a group-based parenting intervention aimed at improving behavioral outcomes among children living in poverty. Their intervention was associated with a cost of $178,000 per child and a cost of $91,100 per child, both in 2008 dollar, for severe behavioral problems avoided and for attention-deficit hyperactivity disorder avoided, respectively. These can be converted into 2019 dollar at around $211,000 and $108,000, which are substantially higher than the ICER in our RLPL program. It is worth noting that as the two programs focused on different outcomes, exact comparisons between our study and the one conducted by Corso et al. (2015) cannot be made, but it may still help to illustrate the magnitude of cost-effectiveness, and to aid stakeholders’ investment decisions across programs. In addition, other research with a similar school readiness intervention (e.g., Head Start REDI) has documented the importance of such evaluations along with how initial costs may pay off years later (Bierman et al., 2018).

The present study extended work in this area by examining cost-effectiveness for the different implementation versions of the RLPL intervention. Results found that costs were low across all implementation methods. In particular, the program could be more cost-effective by using a “train-the-trainer” model, due to its great potential of reaching economies of scale. Future research needs to further investigate the “train-the-trainer” model of implementation as this model is expanded.

### Limitations

Although this study was unique in many respects, we recognize several limitations. First, there is a lack of comparable evidence in the literature that we can use as a reference case to assess our findings. This is mainly because cost-effectiveness analyses on early-childhood self-regulation have been sparse. However, this provides us with a unique opportunity to address this critical knowledge gap, and sets up a model for future cost-effectives studies to compare with, which we see as the major contribution of our current study. In addition, we collected data from previous interventions of our program and conducted a retrospective cost-effectiveness analysis in this study. In so doing, we could only look back at how the intervention was already implemented in each model, and assess the cost and effectiveness that were already in place. As a result, we encountered some uncertainty and variance in parameters. To address this limitation, we conducted a series of sensitivity analyses aiming to adjust for various possibilities. Our sensitivity analyses also provide evidence to support stakeholders’ decisions on replicating and expanding our interventions under different scenarios and at various scales. Although fidelity data were collected across each study, each model had different levels of resources to monitor and support fidelity of implementation over time and thus, these analyses were not included as part of the present study. Future studies should consider the relation between cost-effectiveness and fidelity data. Finally, we propose that future studies should analyze cost-effectiveness on early childhood interventions prospectively as part of the development and implementation process. This approach will empower researchers to evaluate interventions alongside intervention implementation, so that cost and effectiveness data will be better aligned with study designs and have greater potential to inform practice and policy.

### Conclusion

Our economic evaluation of RLPL fills an important gap related to the value of investment in early childhood self-regulation interventions. These results can serve as a model for future cost-effectiveness analyses of early childhood intervention programs and inform policymakers and stakeholders on the cost-effectiveness for the potential impact of an intervention delivered by different models, so that resource allocation and future program design can be optimized.

## Data Availability Statement

The original contributions presented in the study are included in the article/supplementary material, further inquiries can be directed to the corresponding author.

## Ethics Statement

The studies involving human participants were reviewed and approved by Oregon State University Institutional Review Board. Written informed consent to participate in this study was provided by the participants’ legal guardian/next of kin.

## Author Contributions

All authors contributed to the conceptualization and design of the study, data analysis and results, and the writing of the manuscript.

## Funding

The research reported here was supported by the U.S. Department of Education Institute for Education Sciences grants # R305A150196 (PI: McClelland) to Oregon State University. The content is the responsibility of the authors and does not necessarily represent the official views of the Institute of Education Sciences, or the U.S. Department of Education. This research was supported by grants from the Ford Family Foundation and Oregon State University.

## Conflict of Interest

The authors declare that the research was conducted in the absence of any commercial or financial relationships that could be construed as a potential conflict of interest.

## Publisher’s Note

All claims expressed in this article are solely those of the authors and do not necessarily represent those of their affiliated organizations, or those of the publisher, the editors and the reviewers. Any product that may be evaluated in this article, or claim that may be made by its manufacturer, is not guaranteed or endorsed by the publisher.
